# High risk of hepatic complications in kidney transplantation with chronic hepatitis C virus infection

**DOI:** 10.1038/s41598-025-15169-4

**Published:** 2025-08-10

**Authors:** Shih-Ting Huang, Ya-Wen Chuang, Chih-Wei Chiu, Kuo-Ting Sun, Shih-Sheng Chang, I-Kuan Wang, Brain K. Lee, Shiang-Cheng Kung, Chi-Yuan Li, Tung-Min Yu

**Affiliations:** 1 Division of Nephrology, Taichung Veterans General Hospital, Taiwan Boulevard Sect. 4, Taichung, 40705 Taiwan; 2 Department of Post-Baccalaureate Medicine, College of Medicine, National Chung Hsing University, Taichung, Taiwan; 3 College of Medicine, China Medical University, Taichung, Taiwan; 4 Graduate Institute of Biomedical Sciences, School of Medicine, College of Medicine, China Medical University, Taichung, Taiwan; 5 Department of Applied Mathematics and Institute of Statistics, National Chung Hsing University, Taichung City, 402 Taiwan; 6 Dell Seton Medical Center, University of Texas, Austin, USA; 7 Department of Medicine, Division of Nephrology, UCSF Medical Center, San Francisco, CA USA

**Keywords:** Hepatitis C virus, End-stage renal disease, Kidney transplantation, Dialysis, Hepatic disease, Medical research, Nephrology

## Abstract

Data on liver issues including liver cirrhosis, hepatocellular carcinoma, and hepatic failure in renal transplant patients with HCV infection are scarce. In the present study, we conducted a large-scale population-based analysis to investigate the long-term outcomes of renal recipients with HCV infection. Propensity score matching with a ratio of 1:1 was applied. A total of 6,473renal recipients with HCV infection in case group were enrolled after PSM. Our findings showed that subjects with HCV infection in kidney transplant had significantly higher risk of hepatoma, cirrhosis, hepatic failure, and overall hepatic disease than those without HCV infection. (hepatoma: HR: 8.957; 95% CI: 5.324–15.069; cirrhosis: HR: 5.378; 95% CI: 4.363–6.631; hepatic failure: HR: 3.258; 95% CI: 2.527-4.200; overall hepatic disease: HR: 4.128; 95% CI: 3.428–4.971). In the present study, our findings show that renal recipients with HCV infection is significantly associated with a remarkably high risk of hepatic complications post-kidney transplantation.

## Introduction

Hepatitis C virus infection is a global health problem worldwide. It is a well-known entity of liver disease, but HCV infection has also been reported in patients with chronic renal failure. For example, it has been estimated that hepatitis C virus (HCV) has infected approximately 1% of the general population in the US, with rates of up to 3–14% in patients with chronic kidney disease (CKD)^1^.

In dialysis patients, HCV infection rates of 10–65% have been reported and 6–46% of kidney transplant recipients were reported to be infected with HCV, which suggests that overall HCV prevalence may reach as high as 5 times greater in chronic kidney disease compared with the general population^2,3^.

For patients with end-stage renal disease (ESRD), kidney transplantation offers a better quality of life compared to that of patients on dialysis. Kidney transplantation is still considered to be a better treatment for ESRD with HCV infection compared with those remaining on dialysis^4–6^. Hence, HCV infection status in ESRD patients does not affect their suitability to receive kidney transplantation^7–11^.

However, some concerns still exist in renal transplant recipients, including cardiovascular disease, malignancy, and infection risk. HCV infection has been demonstrated to possibly adversely affect long-term outcomes of kidney transplantation because of post-transplant renal allograft nephropathy, post-transplantation diabetes mellitus, and various liver diseases. In HCV renal transplantation, higher risk of post-transplant glomerulonephritis was reported in renal recipients^12^. Additionally, a pyramid of evidence suggests that post-transplant diabetes is significantly associated with cardiovascular events in renal recipients and that HCV has also been associated with a higher risk for insulin resistance and diabetes mellitus post-transplantation^5^. Moreover, hepatic complications are still a critical concern in HCV recipients after transplantation including liver cirrhosis, hepatocellular carcinoma (HCC), and hepatic failure. In cases who develop tumor or cirrhosis caused by the detrimental effect of HCV, a much longer period of time is warranted after transplantation^13^. Data on liver issues in renal transplant patients with HCV infection are scarce and most previous studies have been limited by very small sample sizes without a long observational period, limited comorbidity data, and in particular a lack of information on HCV sero-status in registry datasets, as well as a lack of comparable control cases^4^. In the present study, we conducted a large-scale population-based analysis to investigate the long-term outcomes of renal recipients with HCV infection.

## Methods and materials

Our study population was drawn from 89 Healthcare Organizations (HCOs), comprising 195,071 transplant individuals. Patients who received kidney transplantation [ICD-10: Z94.0] and had been diagnosed with HCV before kidney transplantation served as the case group in this study, while those who were not diagnosed with HCV before kidney transplantation served as the control group. Propensity score matching with a ratio of 1:1 was applied, which was based on sex, age at index, and comorbidities.

### Data source

TriNetX is a multicenter federated health research network that includes various healthcare institutions, such as academic medical centers, specialty physician practices, and community hospitals. They provide electronic health records (EHR) data, which are anonymized to protect identifiable patient health information, and ensure that the data remains de-identified in all circumstances. As a federated network, research studies using TriNetX do not require ethical approval or patient informed consent since no identifiable information is received. The specific database we utilized within TriNetX is the Research Network. The available data in this database include demographics and diagnoses, which are coded using ICD-10 (International Classification of Diseases, 10th edition).

### Study population

Our study population was drawn from 89 Healthcare Organizations (HCOs), comprising 195,071 transplant individuals. Patients who received kidney transplantation [ICD-10: Z94.0] and had been diagnosed with HCV before kidney transplantation served as the case group in this study, while those who were not diagnosed with HCV before kidney transplantation served as the control group. Propensity score matching with a ratio of 1:1 was applied, which was based on sex, age at index, and comorbidities. Participants who were aged < 18 years were eliminated from the study.

### Main outcome and covariates

There were seven outcomes in this study, which were all defined at least one year follow-up time after the index event. The outcomes of interest were:

1. Death.
2. Overall hepatic disease: Liver cell carcinoma (ICD-10: C22.0), Liver (ICDO3: C22.0), Fibrosis and cirrhosis of liver (ICD-10: K74), Hepatic failure, not elsewhere classified (ICD-10: K72).
3. Hepatoma: Liver cell carcinoma (ICD-10: C22.0), Liver (ICDO3: C22.0).
4. Cirrhosis: Unspecified cirrhosis of liver (ICD-10: K74.60), Hepatic fibrosis (ICD-10: K74.0).
5. Hepatic failure (ICD-10: K72).
6. Graft failure: Dependence on renal dialysis (ICD-10: Z99.2), Creatinine [Mass/volume] in Serum, Plasma or Blood (at least 6.00 mg/dL) (TNX:9024), Glomerular filtration rate/1.73 sq M.predicted [Volume Rate/Area] in Serum, Plasma or Blood by Creatinine-based formula (MDRD) (at most 5.00 mL/min/{1.73_m2}(TNX:8001), Glomerular filtration rate/1.73 sq M.predicted [Volume Rate/Area] in Serum, Plasma or Blood by Creatinine-based formula (CKD-EPI) (at most 5.00 mL/min/{1.73_m2} (LNC:62238-1).
7. Rejection (ICD-10: T86.11): Furthermore, we incorporated some covariates in our study, such as age, sex, race, ethnicity, some related comorbidities, and medications. The related comorbidities included hypertension (ICD-10: I10-I15), heart failure (ICD-10: I50), type 2 diabetes mellitus (ICD-10: E11), overweight and obesity (ICD-10: E66), glomerular diseases (ICD-10: N00-N08), and Unspecified viral hepatitis B (ICD-10: B19.1). For medications, we included glucocorticoids tacrolimus, mycophenolate mofetil, mycophenolic acid, and basiliximab. The relevant comorbidities and medications were defined within 10 years before the index date.

### Statistical analyses

Baseline categorical variables were assessed by chi-square test, and the difference of mean age and follow-up time was estimated by the Student’s t test. HRs with 95% CIs were calculated using a proportional hazard model wherein the cohort to which the patient belonged was used as the independent variable. The proportional hazard assumption was tested using the generalized Schoenfeld approach built in the TriNetX platform. Survival probability was demonstrated using Kaplan–Meier’s survival curve and the log-rank test was also used to calculate the difference. All analyses were performed on the TriNetX platform. The statistical significance level was set at a two-sided p-value of < 0.05. Additionally, differences in all variables between the two cohorts were also compared by the standardized mean difference (SMD). If the SMD value was less than 0.1, the difference between the two cohorts was considered negligible.

**Fig. 1:**
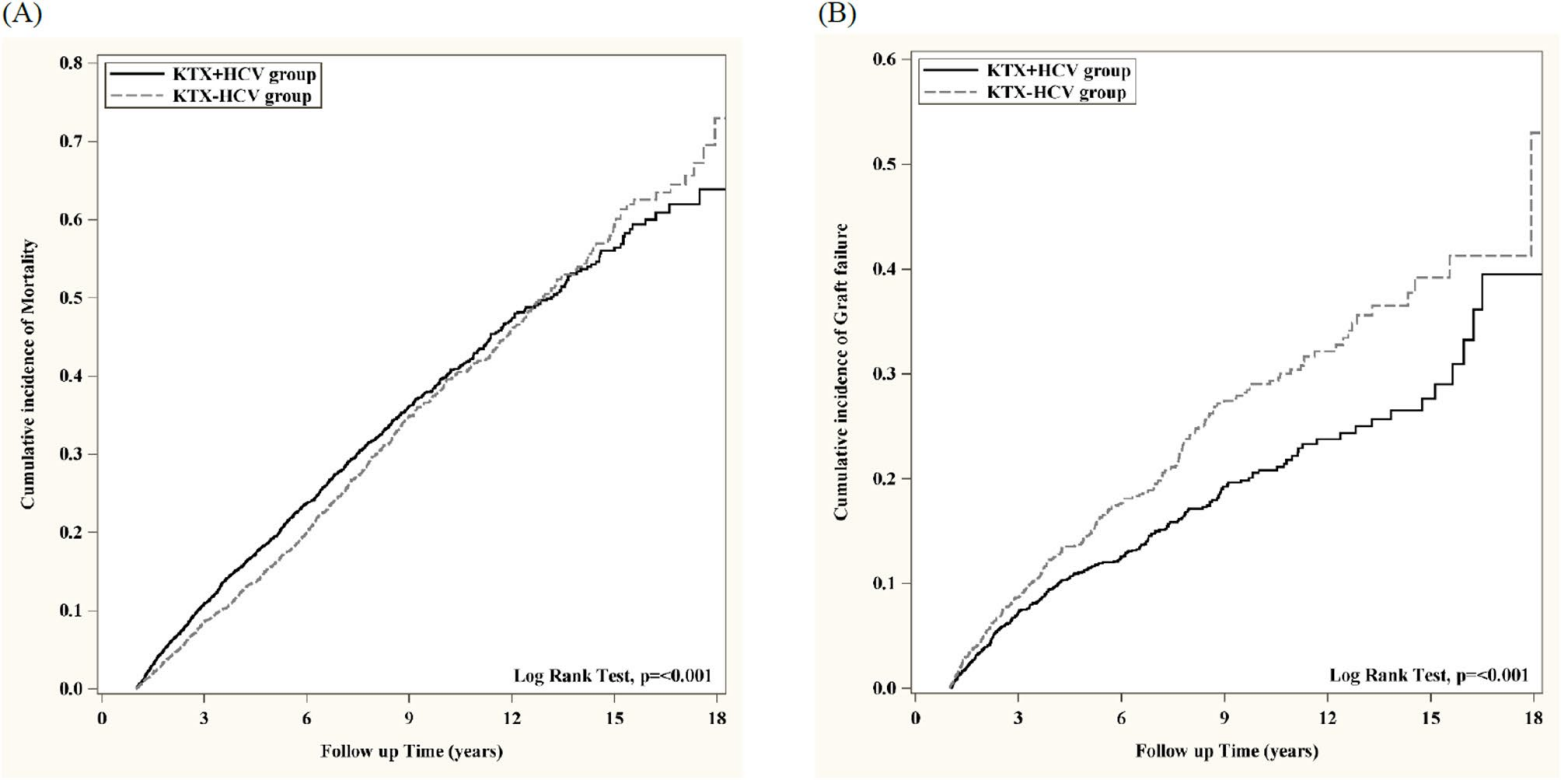
Comparisons of the patient and renal allograft survival between groups with and with chronic hepatitis C virus infection in panel A and B, respectively and that reached the statistical significance (*p* < 0.001).

**Fig. 2:**
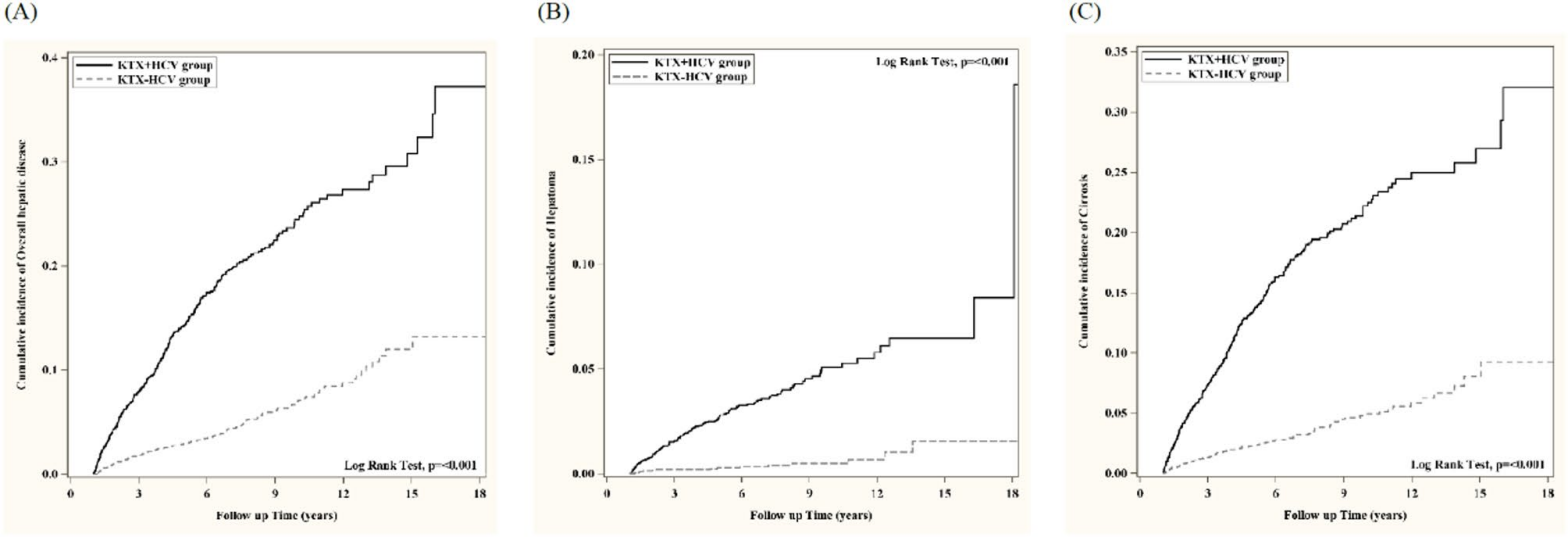
Comparison of overall hepatic disease (A), hepatoma (B) and cirrhosis (C) between renal recipients with and without chronic hepatitis c virus infection in panel A, B and C, respectively. A high risk of overall hepatic disease, hepatoma and cirrhosis was noted in renal recipients with HCV which reached statistical significance (*p* < 0.001).

## Results

Table 1 shows the demographic characteristics and comorbidities of the case and control groups before and after propensity score matching. In the study population, over 60% of the patients were male and aged 40–65 years. Additionally, we found that the majority of the study population was not Hispanic or Latino. After matching, the differences in demographic characteristics and comorbidities between the two groups were small and well matched.

**Table 1:** Demographic and comorbidity comparison of kidney transplant recipients with and without hepatitis C virus infection before and after propensity score matching.

Demographics	Before propensity score matching				After propensity score matching			
	HCV	Non-HCV			HCV	Non-HCV		
	*N* (%)	*N* (%)	*p*-value	Std diff.	*N* (%)	*N* (%)	*p*-value	Std diff.
Age								
18–40 years	344(5.30%)	33150(18.54%)	< 0.0001	0.4172	344(5.31%)	358(5.53%)	0.5869	0.0096
40–65 years	4399(67.81%)	99991(55.92%)	< 0.0001	0.2468	4390(67.82%)	4391(67.84%)	0.985	0.0003
65 + years	1741(26.84%)	41721(23.33%)	< 0.0001	0.081	1736(26.82%)	1721(26.59%)	0.7657	0.0052
Sex								
Male	4434(68.35%)	100450(56.17%)	< 0.0001	0.2533	4425(68.36%)	4423(68.33%)	0.9699	0.0007
Female	1803(27.79%)	72618(40.61%)	< 0.0001	0.2726	1801(27.82%)	1797(27.76%)	0.9374	0.0014
Race								
Hispanic or Latino	741(11.42%)	19084(10.67%)	0.0546	0.024	741(11.45%)	715(11.05%)	0.4695	0.0127
Not Hispanic or Latino	4251(65.53%)	116372(65.08%)	0.4496	0.0096	4243(65.55%)	4288(66.24%)	0.4041	0.0147
White	2885(44.47%)	92111(51.51%)	< 0.0001	0.1412	2880(44.49%)	2842(43.91%)	0.5013	0.0118
Black or African American	1972(30.40%)	38757(21.67%)	< 0.0001	0.1998	1968(30.40%)	1997(30.85%)	0.5803	0.0097
Asian	243(3.75%)	8870(4.96%)	< 0.0001	0.0595	243(3.75%)	250(3.86%)	0.7479	0.0057
Comorbidities								
Hypertensive diseases	5277(81.35%)	85062(47.57%)	< 0.0001	0.7543	5263(81.31%)	5346(82.59%)	0.0579	0.0333
Type 2 diabetes mellitus	3245(50.02%)	42072(23.53%)	< 0.0001	0.5715	3236(49.99%)	3249(50.19%)	0.8192	0.004
Overweight and obesity	1384(21.34%)	19976(11.17%)	< 0.0001	0.2782	1377(21.27%)	1355(20.93%)	0.6356	0.0083
Heart failure	1422(21.92%)	16996(9.50%)	< 0.0001	0.3463	1420(21.94%)	1445(22.32%)	0.5966	0.0093
Glomerular diseases	900(13.87%)	18171(10.16%)	< 0.0001	0.1144	899(13.89%)	899(13.89%)	1	< 0.0001
Viral hepatitis B	436(6.72%)	936(0.52%)	< 0.0001	0.3364	422(6.52%)	378(5.84%)	0.1083	0.0282
Glucocorticoids	3921(60.44%)	63090(35.28%)	< 0.0001	0.5205	3907(60.36%)	3948(60.99%)	0.4607	0.013
Tacrolimus	2721(41.95%)	34521(19.30%)	< 0.0001	0.5067	2707(41.82%)	2709(41.85%)	0.9716	0.0006
Mycophenolate mofetil	2232(34.41%)	27279(15.25%)	< 0.0001	0.4546	2220(34.30%)	2204(34.05%)	0.7669	0.0052
Mycophenolic acid	897(13.83%)	17364(9.71%)	< 0.0001	0.128	895(13.83%)	916(14.15%)	0.5947	0.0094
Basiliximab	559(8.62%)	4548(2.54%)	< 0.0001	0.267	555(8.57%)	551(8.51%)	0.8999	0.0022

We observed that before matching, the number of individuals in each age group varied between the HCV and non-HCV groups (age 18–40: 344 vs. 33150 / age 40–65: 4399 vs. 99991 / age > 65: 1741 vs. 41721). After matching, the number of individuals in each age group tended to be similar between the two groups (age 18–40: 344 vs. 358 / age 40–65: 4390 vs. 4391 / age > 65: 1736 vs. 1721). Furthermore, the number of individuals in each gender also varied between the HCV and non-HCV groups (male: 4434 vs. 100450 / female: 1803 vs. 72618). However, after matching, the number of males and females in the HCV and non-HCV groups also tended to be similar (male: 4425 vs. 4423 / female: 1801 vs. 1797).

As Table 2 shows, subjects with HCV after receiving a kidney transplant had significantly higher risk of death, hepatoma, cirrhosis, hepatic failure, and overall hepatic disease than those without HCV after receiving a kidney transplant in this study cohort (death: HR: 1.19; 95% CI: 1.098–1.290; *P* =<0.001 / hepatoma: HR: 8.957; 95% CI: 5.324–15.069; *P* =<0.001 / cirrhosis: HR: 5.378; 95% CI: 4.363–6.631; *P* =<0.001 / hepatic failure: HR: 3.258; 95% CI: 2.527-4.200; *P* =<0.001 / overall hepatic disease: HR: 4.128; 95% CI: 3.428–4.971; *P* =<0.001). However, subjects with HCV after receiving a kidney transplant had significantly lower risk of graft failure and rejection than those without HCV after receiving a kidney transplant in this study cohort (graft failure: HR: 0.696; 95% CI: 0.597–0.812; *P* =<0.001 / rejection: HR: 0.696; 95% CI: 0.598–0.807; *P* =<0.001). As shown in Table 3, before PSM, among female or male patients, the risk of death in patients who had been diagnosed with HCV after receiving a kidney transplant was significantly higher than that of the non-HCV patients after receiving a kidney transplant (female: aHR: 1.448; 95%CI: 1.300-1.612 / male: aHR: 1.406; 95%CI: 1.319–1.499). Similarly, patients aged 18 to 40 years old and 40 to 65 years old showed the same trend (age 18–40: aHR: 4.084; 95%CI = 2.651–6.293 / age 40–65: aHR: 1.663; 95%CI = 1.502–1.842). Furthermore, regardless of whether the subjects were male or female, the risk of Overall hepatic Disease in patients who had been diagnosed with HCV after receiving a kidney transplant was significantly higher than in the non-HCV group (female: aHR: 4.832; 95%CI: 3.865–6.041 / male: aHR: 5.692; 95%CI: 4.985–6.499). In patients of different age groups receiving a kidney transplant, the results also showed that the risk of Overall hepatic Disease was significantly higher in patients who had been diagnosed with HCV receiving kidney transplant compared to those not diagnosed with HCV after the transplant (age 18–40: aHR: 6.184; 95%CI = 2.755–13.879 / age 40–65: aHR: 5.104; 95%CI = 4.206–6.193/ age > 65: 5.017; 95%CI = 4.384–5.742).

**Table 2:** Hazard ratio and 95% confidence interval for risk estimates of patient, renal allograft and hepatic outcomes in both groups before/after propensity scoring matching.

	Before PSM					After PSM				
	HCV		Non-HCV			HCV		Non-HCV		
	Number	Events	Number	Events	Hazard Ratio (95% CI)	Number	Events	Number	Events	Hazard Ratio (95% CI)
Death	6,000	1,367	170,861	27,204	1.43(1.354, 1.509)***	5,986	1,367	6,084	1,050	1.19(1.098, 1.290)***
Graft failure	2,859	317	97,288	14,325	0.697(0.623, 0.779)***	2,853	316	2,469	338	0.696(0.597, 0.812)***
Rejection	5,337	315	149,778	14,407	0.561(0.502, 0.628)***	5,326	315	5,147	373	0.694(0.598, 0.807)***
Hepatoma	5,061	126	177,598	356	12.12(9.891, 14.852)***	5,054	126	6,319	16	8.957(5.324, 15.069)***
Cirrhosis	2,794	327	171,165	2,795	7.418(6.615, 8.319)***	2,790	326	5,736	120	5.378(4.363, 6.631)***
Hepatic failure	4,147	211	172,363	2,003	4.261(3.697, 4.910)***	4,142	211	5,830	83	3.258(2.527, 4.200)***
Overall hepatic disease	2,539	329	168,654	4,158	5.425(4.849, 6.070)***	2,536	329	5,585	168	4.128(3.428, 4.971)***

**Table 3:** Hazard ratios and confidence intervals of patient death and overall hepatic complications in kidney transplant recipients with and without hepatitis virus C infection before and after propensity score matching, stratified by all variables.

	Before PSM					After PSM				
	HCV		Non-HCV			HCV		Non-HCV		
	Patients in cohort	Patients with outcome	Patients in cohort	Patients with outcome	Hazard Ratio (95% CI)	Patients in cohort	Patients with outcome	Patients in cohort	Patients with outcome	Hazard Ratio (95% CI)
Death										
Gender										
Male	4,100	996	95,553	16,307	1.406(1.319, 1.499)	4,094	996	4,164	762	1.198(1.091, 1.317)
Female	1,660	344	69,729	10,372	1.448(1.300, 1.612)	1,653	343	1,697	288	1.101(0.941, 1.288)
Age										
18–40	162	21	23,322	1,086	4.084(2.651, 6.293)	159	21	172	11	2.408(1.160, 4.998)
40–65	2,347	385	83,325	8,811	1.663(1.502, 1.842)	2,341	384	2,421	271	1.394(1.193, 1.629)
> 65	3,813	1,029	69,607	18,064	1.029(0.967, 1.096)	3,791	1,020	3,782	957	0.974(0.892, 1.064)
Hypertensive diseases										
Yes	5,003	1,124	83,909	12,912	1.398(1.316, 1.486)	4,987	1,120	5,082	927	1.096(1.005, 1.196)
No	995	243	169,808	27,124	1.251(1.102, 1.419)	994	243	1,028	186	1.052(0.869, 1.274)
Type 2 diabetes mellitus										
Yes	3,046	748	41,033	8,067	1.136(1.054, 1.225)	3,028	743	3,071	629	1.106(0.995, 1.230)
No	2,945	618	169,808	27,124	1.183(1.092, 1.281)	2,941	616	2,975	530	0.993(0.884, 1.115)
Viral hepatitis B										
Yes	576	108	1,326	192	1.217(0.961, 1.541)	544	103	570	88	0.991(0.744, 1.318)
No	5,424	1,259	169,547	27,015	1.455(1.375, 1.539)	5,424	1,259	5,487	1,000	1.116(1.027, 1.213)
Overall hepatic disease										
Gender										
Male	1,734	241	94,623	2,374	5.692(4.985, 6.499)	1,734	241	3,804	110	4.649(3.710, 5.825)
Female	750	81	68,807	1,596	4.832(3.865, 6.041)	748	80	1,571	50	3.15(2.211, 4.486)
Age										
18–40	105	10	22,738	323	6.184(2.755, 13.879)	104	10	152	10	5.351(1.077, 26.587)
40–65	1,058	109	81,333	1,786	5.104(4.206, 6.193)	1,057	109	2,168	53	4.211(3.032, 5.847)
> 65	1,517	234	69,792	2,179	5.017(4.384, 5.742)	1,513	234	3,541	142	3.712(3.013, 4.573)
Hypertensive diseases										
Yes	2,191	268	81,217	2,115	4.697(4.136, 5.333)	2,188	268	4,679	141	3.841(3.132, 4.709)
No	347	61	167,594	4,143	6.361(4.940, 8.190)	347	61	961	25	5.756(3.610, 9.178)
Type 2 diabetes mellitus										
Yes	1,178	138	39,321	1,189	3.931(3.296, 4.689)	1,178	138	2,810	83	3.98(3.031, 5.225)
No	1,359	191	167,594	4,143	5.253(4.543, 6.073)	1,359	191	2,810	74	4.652(3.556, 6.085)
Viral hepatitis B										
Yes	164	20	923	79	1.261(0.771, 2.062)	162	19	355	27	1.224(0.680, 2.206)
No	2,375	309	167,737	4,079	5.57(4.962, 6.254)	2,375	309	5,233	129	4.989(4.063, 6.127)

After PSM, it can be seen that in male patients, the risk of death was significantly higher in those diagnosed with HCV after receiving a kidney transplant compared to those not diagnosed with HCV after the kidney transplant (male: aHR: 1.198; 95%CI: 1.091–1.317). Patients aged 18 to 40 years old and 40 to 65 years old also showed the same results as male patients (age 18–40: aHR: 2.408; 95%CI = 1.160–4.998 / age 40–65: aHR: 1.394; 95%CI = 1.193–1.629). When we examined Overall hepatic Disease, prevalence rates in patients who had been diagnosed with HCV after receiving a kidney transplant were significantly higher than those of the non-HCV cohort (female: aHR: 3.150; 95%CI: 2.211–4.486 / male: aHR: 4.649; 95%CI: 3.710–5.825). Additionally, regardless of age group the risk of Overall hepatic Disease was significantly higher in patients who had been diagnosed with HCV after receiving a kidney transplant compared to those who had not been diagnosed with HCV after the transplant (age 18–40: aHR: 5.351; 95%CI = 1.077–26.587 / age 40–65: aHR: 4.211; 95%CI = 1.502–1.842/ age > 65: 3.712; 95%CI = 3.013–4.573).

Furthermore, most of the comorbidity results indicated that regardless of whether patients had comorbidities, those diagnosed with HCV after receiving a kidney transplant had a significantly higher risk of death and Overall hepatic Disease compared to those not diagnosed with HCV after the transplant.

The Kaplan-Meier curve of survival probability of death, renal allograft are shown in Fig. 1A, B and overall hepatic disease including hepatoma, cirrhosis and hepatic failure in Fig. 2 A, B, C, respectively after PSM selection.

## Discussion

In the present 10-year observational study, associated confounding factors including age, transplant year, sex, and comorbidities, such as hypertension, diabetes mellitus, obesity, primary glomerulonephritis and immunosuppressant agents, were included in a large-scale analysis of a HCV renal recipient cohort that was propensity score-matched with a non-HCV renal cohort. Our findings showed that there was only a slightly higher mortality risk among renal recipients with HCV infection, with a 1.19-fold increased mortality risk. In a previous study, a high mortality and graft failure risk was reported in HCV-infected renal recipients. HCV renal recipients were thus presumed to have a greater likelihood of having an unfavorable outcome after kidney transplantation^14^. Moreover, a higher risk for death was found during the first 6 months after transplant. However, transplantation still had a superior outcome compared with remaining on dialysis in terms of long-term outcomes in an investigation of patients receiving either transplantation or continuing to receive hemodialysis^15^. In the present study, we directly compared renal recipients with and without HCV infection, and the findings showed only a slightly increased risk of mortality in HCV infection, suggesting a significant improvement in post-transplantation care for HCV-infected renal recipients. Moreover, our findings showed a favorable survival of renal allograft in HCV infection with a significantly lower risk of graft failure (0.696-fold decrease). This result is consistent before and after propensity score matching, which reached statistical significance. In previous studies, the risk associated with renal allograft loss was remarkably attenuated in multivariable models that included associated confounders, such as longer duration of waiting list, higher PRA, and re-transplantation^16^. Our findings support results reported in previous studies.

In kidney transplantation with HCV infection, whether HCV infection could increase the risk of acute rejection remains considerably controversial^5,14^. ,^17–20^ In the previous literature, the results were conflicting because certain data, including PRA level, waiting time, and immunosuppressive agents, such as induction therapy, were not assessed, and many studies had small case numbers with a short study period^6^. In the present study, our results showed a lower risk of renal allograft rejection in HCV-infected recipients. There was a 0.561-fold decreased risk before PSM and 0.694-fold decreased risk after PSM, which both reached statistical significance. These findings are notable as they have never been reported previously. In the case of HCV-infected renal recipients, a previous study suggested that a decreased number of naive T helper lymphocytes, as well as low responses of T helper lymphocytes to stimulation by mitogens would result in an immunodeficient state and that may account for the decreased acute rejection risk in HCV-infected renal recipients^6,21^. Our results appear to provide solid evidence supporting these findings. Taken together, in comparison to the renal recipients without HCV infection, a distinct improvement of renal allograft survival with a lower graft failure risk and a low rejection risk was noted in this study. The application of modern immunosuppression and superior post-transplant care may account for the better renal allograft results in this study.

Hepatic complications remain a major concern in renal recipients with chronic HCV infection and data on the cause-specific risks of liver cirrhosis, hepatoma, and liver failure post-transplantation remain lacking. Previous studies suggested that HCV-infected recipients had higher death rates caused by liver disease than HCV-negative patients in a univariate analysis^22–25^. In the present study, multiple relevant variables were included to eliminate bias to the greatest extent possible through propensity score matching. A comparison of renal recipients groups with and without HCV infection demonstrated that HCV-infected recipients were significantly associated with a remarkably high risk of hepatic complications post-kidney transplantation. A 5.425-fold increased risk was noted before PSM, which was consistent with a 4.128-fold risk after PSM. Moreover, a 12.12-fold increased risk of new onset hepatoma post-kidney transplantation was observed before PSM and there was an 8.957-fold greater risk after PSM. With respect to other hepatic complications, including liver cirrhosis as well as hepatic failure, the trend was consistent and was significantly different. To the best of our knowledge, this is the first study to report the cause-specific risk ratio of post-transplantation hepatic disease, including hepatoma, cirrhosis, and liver failure in renal recipients with HCV infection. To date, the hepatoma risk in HCV-infected recipients has been neglected and data on the hepatoma risk post-transplantation in HCV-infected recipients remain lacking. In one study with a small case number, very few isolated cases of hepatocellular carcinoma (HCC) were reported. It has been proposed that the risk of developing chronic liver disease post-transplantation is closely associated with duration of disease, possibly over 20 years^26^. A high risk of HCC post-transplantation was observed in the study, which reached a 8.957-fold increased risk in HCV-infected transplantation and this finding has never been reported previously. Our findings indicate that hepatic complications after kidney transplantation remain a major concern in renal recipients with HCV infection, which has to date been neglected.

Our study was focused on hepatic outcomes and did not explore non-hepatic post-transplant complications (e.g., cardiovascular events, new-onset diabetes), which are also clinically important and warrant further investigation in future studies.

Recently, direct-acting antivirals (DAAs) have been demonstrated to dramatically transform the care of patients with chronic hepatitis C virus (HCV) infection^3,27–30^. In kidney transplantation, DAAs have been demonstrated to remarkably reduce the risk of death after renal transplantation and that early commencement of DAAs post-transplantation would improve patient and allograft survival among HCV-positive recipients^3^. Because a high risk of hepatic complication has been demonstrated, early eradication of HCV infection is recommended for patients requiring renal transplantation^17^.

## Limitations

While, the results presented herein are robust, there were some limitations in this study. First, some clinical data associated with renal transplant recipients could not be obtained. For example, although the important etiology of acute rejection could be identified in the database, other findings regarding renal allograft biopsy and relevant immunological data could not be accessed to further clarify the etiology of renal allograft failure, such as post-transplant glomerulonephritis related to HCV, Bk virus nephropathy, chronic pyelonephritis and CNI nephropathy. Second, because of the observational nature of this study, some inherent bias may have existed despite the inclusion of associated confounders in the analysis of propensity score matching (PSM).

## Conclusion

Improvements of patient and renal allograft survival were noted in renal recipients with HCV infection. However, post-transplantation liver complications remain a major concern in renal recipients with HCV infection. Further research on renal recipients with HCV is warranted, particularly with respect to the role of DAA, which is a highly effective treatment in HCV infection.

## Data Availability

Data from the TriNetX database is not publicly available. However, the data of this study are openly accessible upon reasonable request to the TriNetX administrators through their website ( https://trinetx.com) as well as that the corresponding author may also be contacted.

## Abbreviations

HCV: Hepatitis C virus
CKD: Chronic kidney disease
ESRD: End stage renal disease
HCC: Hepatocellular carcinoma
ICD-10-CM: International classification of diseases, 10th edition, clinical modification
EHR: Electronic health records
CIs: Confidence intervals
SMD: Standardized mean difference
HR: Hazard ratio
PSM: Propensity score matching
PRA: Panel reactive antibodies
